# Artificial neural networks reveal individual differences in metacognitive monitoring of memory

**DOI:** 10.1371/journal.pone.0220526

**Published:** 2019-07-31

**Authors:** Alexandria C. Zakrzewski, Matthew G. Wisniewski, Helen L. Williams, Jane M. Berry

**Affiliations:** 1 Department of Psychological Sciences, Kansas State University, Manhattan, Kansas, United States of America; 2 School of Psychology, Keele University, Keele, England; 3 Department of Psychology, University of Richmond, Richmond, Virginia, United States of America; Fordham University, UNITED STATES

## Abstract

Previous work supports an age-specific impairment for recognition memory of pairs of words and other stimuli. The present study tested the generalization of an associative deficit across word, name, and nonword stimulus types in younger and older adults. Participants completed associative and item memory tests in one of three stimulus conditions and made metacognitive ratings of perceptions of self-efficacy, task success (“postdictions”), strategy success, task effort, difficulty, fatigue, and stamina. Surprisingly, no support was found for an age-related associative deficit on any of the stimulus types. We analyzed our data further using a multilayer perceptron artificial neural network. The network was trained to classify individuals as younger or older and its hidden unit activities were examined to identify data patterns that distinguished younger from older participants. Analysis of hidden unit activities revealed that the network was able to correctly classify by identifying three different clusters of participants, with two qualitatively different groups of older individuals. One cluster of older individuals found the tasks to be relatively easy, they believed they had performed well, and their beliefs were accurate. The other cluster of older individuals found the tasks to be difficult, believed they were performing relatively poorly, yet their beliefs did not map accurately onto their performance. Crucially, data from the associative task were more useful for neural networks to discriminate between younger and older adults than data from the item task. This work underscores the importance of considering both individual and age differences as well as metacognitive responses in the context of associative memory paradigms.

## Introduction

Research has revealed an age-specific impairment for recognition memory of pairs of words and other stimuli [1–5]. Older adults have greater difficulty remembering associations between components of information (e.g., a person’s face and name) than individual components (e.g., a person’s face, a person’s name; [6]). Most investigations of this effect have used an experimental procedure involving item-associative recognition tests (e.g., [7]). For example, after studying a list of word-pairs (associates) such as *wardrobe-ocean*, participants are asked to identify the individual words they studied (e.g., *wardrobe*) as “old” and words they did not study (e.g., *circus*) as “new.” Younger and older adults often perform equally well on such item tests. However, when they are asked to identify pairs (e.g., *wardrobe-summer*) as “old” or “new” in the associative test, both younger and older adults perform worse than they did on the item test. Importantly, older adults show a larger drop in performance on the associative test compared to younger adults. This dissociation is known as the associative deficit hypothesis (ADH; [4]).

Several reasons have been proposed to explain the age-specific associative deficit. First, differences in encoding strategies used within and between age groups could explain the deficit [8–10]. Various strategies can be used during associative recognition tasks, including silently repeating the word pair, visualization, sentence generation, inferring personal relevance, and rhyming [11]. Some of these strategies are more effective for accurate recall or recognition than others. For example, when studying the word-pair, *wardrobe-ocean*, an elaborative encoding strategy might include visualizing a large wooden wardrobe sinking to the bottom of the ocean. The strength of this mental image mediator may predict successful retrieval during the associate test when the participant is presented *wardrobe-summer*, and correctly identifies the word pair as “new” [3]. Some have argued that the age-specific associative deficit is a result of older adults experiencing difficulty merging or binding together various aspects of a word-pair into a single unit during encoding ([4, 12]; see also [13] for a similar argument applied to development of elaboration skill during adolescence). If the unit is weak, nonexistent, or unavailable at retrieval, memory performance will be poorer. This outcome may be more likely in older adults if they are unable or unlikely to use effortful encoding strategies such as elaboration. Younger adults, on the other hand, have the cognitive resources (e.g., working memory capacity) to engage in elaborative encoding, making them more successful at paired-associate memory tasks [14].

Age differences in encoding strategies might explain age differences in associative memory but why do older adults often perform equivalently to younger adults on the item task? To explain this age by test-type interaction effect, researchers have suggested that existing knowledge may be especially helpful to older adults on the item test, boosting their performance above that on the associative task through the creation of new concepts and/or mediators [1, 15–17]. Compared to college-aged students, older adults are likely to have a larger assortment of experiences and knowledge that can support performance in a single-item recognition task [16]. Of course, this benefit would depend on the types of items comprising the study and test lists. Arguably, younger adults may have enough experience to remember common words, and older adults do not *always* perform as well as younger adults on item recognition tasks. In fact, some of the earliest studies in cognitive aging show significant age impairment on single-item recognition tasks (e.g., [18, 19]).

Explanations for age differences in performance on the associative task are hotly debated, with no current consensus [6, 20–22]. The associative deficit, though robust (for review, see [23]), varies in magnitude across studies. Research has shown that different test formats (e.g., four-alternative forced-choice) affect the strength of the deficit (e.g., [8, 24]). For example, older adults perform better on associative recognition tasks when demands on strategic retrieval are reduced [8]. This implies that the age-specific associative deficit is related to processes at retrieval. In fact, Fox et al. [3] found that strength of mediators at encoding did not vary between younger and older adults (cf. [25]) but that only younger adults appeared to benefit from mediator strength at test, by producing fewer false alarms than older adults to recombined pairs of words. Additionally, the deficit is attenuated by manipulating both encoding and retrieval instructions, for example, when creating a sentence including the two words [26]. Thus, receiving instructions that support processing during study and test reduces the associative deficit in older adults (also, see [27, 28]).

Furthermore, the deficit seems to depend on type of stimuli used in the memory task. Age-related associative deficit patterns have been found for name–face pairs [16, 29], picture pairs [30], semantic pairs (e.g., occupation) of faces and names [31], and face–spatial location pairs [32]. In contrast, the deficit is absent for non-words (e.g., *bligma*, *lossens*) [1], and proper name pairs [2]. Badham and Maylor [1] suggest that because pre-existing knowledge cannot be used to remember non-word stimuli, younger and older adults rely on familiarity-based processing, leading them to perform similarly on item and associative tasks. A related argument has been made for name-name word pairs, because, like non-word pairs, names carry little inherent semantic structure by which to support creating elaborative or imagery-based associations between two proper names [2]. As a result, younger adults perform similarly to older adults on name-pair retrieval tasks. Recently, McGillivray and Castel [33] found age-equivalent memory performance when word pairs were related, suggesting prior knowledge diminished the age-related memory deficit (e.g., [34]).

Together, these studies suggest that the age-specific associative deficit may be task- and stimulus- specific, and may depend on whether encoding and/or recollection is supported by test format and instruction. These characteristics are properties of the to-be-learned material. However, characteristics of the learner are brought to bear in learning situations as well. As discussed earlier, differences in strategy use at encoding and retrieval affect memory outcomes. Strategy use does not occur in a vacuum but rather is deployed by the learner as the situation demands it. For example, while studying for a vocabulary exam in French 101, a student may monitor her ongoing learning and, based on feedback collected during study (e.g., testing one’s knowledge with flashcards or quiz questions), she may decide to change or continue using her study strategy (e.g., use of mnemonics, visualization, rhyming, etc.). A student who monitors her studying behavior by assessing progress towards a learning goal and shifts strategies accordingly is demonstrating metacognition, that is, monitoring and control of cognitive processes during learning [35–38]. Research shows that strategy use and metacognitive beliefs affect learning and memory outcomes generally, and in research focusing specifically on metacognitive aging (for review, see [11; 39–41]). One purpose of the present study was to apply our knowledge of current research on metacognitive aging to the age-specific associative deficit by analyzing interrelationships between subjective reports of item and associative memory experiences before study and after test.

By analyzing metacognitive processing at study and test, researchers might uncover underlying cognitive mechanisms tied to associative memory and its failure. Metacognitive mechanisms might explain why older adults don’t always exhibit an associative deficit. Different types of tasks, instructions, and stimuli may support the use and effectiveness of metacognitive monitoring and control processes. For example, individuals may deploy different memory strategies when studying items versus associates. Additionally, metacognitive confidence judgements, pre-test and post-test performance predictions, and perceived ratings of strategies used, effort expended, and task experience (e.g., difficulty), may vary between item and associative memory tasks, and *should* vary given the different task demands associated with learning and remembering items versus pairs. Thus, we might expect individuals to provide higher judgments of confidence for learning items than pairs.

Some researchers have posited metamemory (i.e., metacognition in the memory domain) as a predictor of the age-specific associative memory deficit (e.g., [42, 43]) but few have actually asked participants to report metacognitive judgments during tests of the age-related associative deficit. In one study, self-reported metamemory beliefs were significantly related to strategy success and associative memory [14]. In other work, Berry et al. [2] showed that participants appeared to be aware of differences between test type (associative, item). They asked younger and older adults to make judgments about their performance (e.g., *On a scale from 1%-100%*, *how much did you remember in this study*?) after completing associative and item recognition tasks. Overall, these postdictions were higher for the item test than for the associative test, indicating good metacognitive tracking of actual recognition patterns. However, analyses of age differences showed that younger adults made accurate postdiction ratings on both item and associative tests, but that older adults’ postdiction ratings were accurate only on the associative test (they underestimated their performance on the item test). Relatedly, Hertzog, Kidder, Powell-Moman, and Dunlosky [44] found aging did not affect metacognitive monitoring during encoding, despite a decline in associative memory. These results challenge the suggestion that impairment in metamemory explains the associative deficit in older adults [42, 43].

Beyond monitoring accuracy, research that has focused on metacognitive control processes at the strategy-use level sheds some light on the role of metamemory vis-à-vis the associative deficit. Specifically, Bender and Raz [14] found that age-related differences in working memory accounted for a significant portion of variance in recognition of associations and endorsement of shallow strategies (e.g., repetition). Stronger endorsement of effective, deep encoding strategies (e.g., interactive imagery or sentence generation) was linked to more correct and fewer false recognitions of associations. Conversely, belief in inefficient shallow strategies was associated with poor hit rates for items and associations. False beliefs in the efficacy of certain strategies may cause individuals to use ineffective encoding strategies. Relatedly, Berry et al. [2] found that perceived strategy success partially mediated age-related differences on a word recognition associative test.

Together, these findings show that age differences in metacognitive beliefs and use of effective strategies exist but they do not always predict memory performance outcomes. One reason why some studies have failed to show age-related differences in metacognitive measures and, sometimes, in performance (e.g., [45]) is that individual differences exist but might get obfuscated by averaging scores across age groups. For example, within older adult participants, Naveh-Benjamin, Maddox, Jones, Old, and Kilb [46] found that older males but not older females had poorer recognition memory for word pairs, suggesting age-related gender differences in associative memory. Similarly, Amrhein, Bond, and Hamilton [47] found differences within older participants and between older and younger adults in proportion recalled and item-pair clustering of semantic categories (e.g., recalling “dog-cat” and “table-chair” instead of “table-dog” and “cat-chair”) on a free recall task. Specifically, these differences existed between older adults with higher or lower levels of locus of control (LOC; [48]). Interestingly, LOC differences did not moderate younger adults’ recall performance. Thus, individual differences among older adults may need further scrutiny in tests of the associative deficit.

The present study explored effects of aging on metacognition and memory by collecting data on seven metacognitive measures (self-efficacy, postdictions, strategy success, effort, fatigue, difficulty, and stamina) in conjunction with forced-choice yes/no associative and item recognition. First, a planned univariate analysis of the data was conducted on recognition performance to test the associative deficit hypothesis (e.g., [4, 23, 49–52]). Few studies have examined metacognitive processing on item-associative recognition tasks and those that have generally focused on a single metacognitive variable (e.g., JOLs, strategy). Second, to examine patterns in the multivariate data that were related to age differences, a simple artificial neural network was trained to categorize individuals as younger adults or older adults using all dependent variables in the dataset. Unlike many traditional analyses that either require *a priori* groupings of individuals into conditions or collapse across all individuals, multivariate analyses such as artificial neural networks can cluster individuals into groups based on common features that might otherwise go undetected [53]. Here, we explore individual differences in metacognitive memory processes in two age groups, grounded in a data-driven method.

## Methods

### Participants

The University of Richmond IRB approved this research study (for more information, contact Jane Berry—Cognitive Aging Laboratory jberry@richmond.edu). Written informed consent forms were provided and signed by all participants. This experiment utilized a mixed design with Age (Young, Old) and Stimulus Type (Words, Names, Nonwords) as between-subjects factors and Test Type (Item, Associate) as a within-subjects factor. One-hundred and twenty-one participants participated in the study. Fifteen participants (11 older and 4 younger adults) were dropped for not completing all questionnaires. The remaining 106 participants included 49 older adults (30 female) aged 60–82 (*M* = 69.91 years, *SD* = 5.66) and 57 younger adults (35 female) aged 18–22 (*M* = 18.98 years, *SD* = 0.99). Older adults were recruited from the community through newspaper and campus advertisement. Older adults received $20 for participation. Younger adults were students recruited from introductory psychology classes or via campus announcements. Students received either participation credit or $20 for participation. Demographic data and standardized scores on processing speed (measured by the Digit Symbol Substitution Test [54]) and vocabulary (measured by Ekstrom, French, Harman, and Dermen [55] Synonyms Test) for the 106 participants included in analyses are reported in Table 1.

**Table 1:** Means and standard deviations for demographic comparisons between age groups.

Variable	Younger Adults (*N* = 57)	Older Adults (*N* = 49)	*t-*test p values and Cohen’s *d*
Years of education	12.91 (0.96)	16.17 (2.36)	*p* < .001, *d* = 1.96
Self-rated health	8.34 (1.56)	8.63 (1.33)	*ns*
Self-rated vision	8.86 (1.31)	8.55 (1.28)	*ns*
Self-rated hearing	8.86 (1.29)	7.31 (2.41)	*p* < .001, *d* = 0.84
Speed of processing	70.33 (11.20)	48.76 (10.21)	*p* < .001, *d* = 2.02
Vocabulary	22.07 (4.16)	29.06 (4.99)	*p* < .001, *d* = 1.53

*Note*. Scales for self-rated health, vision, and hearing ranged from 0 (poor) to 10 (excellent). Two-tailed *t*-tests were conducted between younger and older adults on each variable. Significant results are reported in the third column.

### Materials

The experimental stimuli were words (common nouns), names (proper first names), and non-words. Names were selected from a Richmond area phone book. Names had 1–3 syllables and a mean length of 5.30 letters (range = 3–11, SD = 1.39). Words and non-words were selected from the English Lexicon Project database [56]. Words had a mean length of 6.46 letters (range = 4–10, SD = 1.14), 1–2 syllables, and were of medium- to high-frequency in the language achieving an average log-transformed HAL frequency of 8.68 [56, 57]. Nonwords were chosen for specific characteristics: None of them had orthographic neighbors. They were all between 6 and 9 letters in length (*M* = 8.21, *SD* = .87). They all had a high probability of being correctly identified as a nonword in a lexical decision task (*M* = .96, *SD* = .03, range = .90–1.00). Also, to ensure the nonwords were not too different from normal words they were selected to have a long reaction time when being judged as nonwords (*M* = 883.11 ms, *SD* = 51.30 ms). Finally, the nonwords were selected to consist of two syllables with each syllable retaining its phonetic characteristics when the two syllables were reversed. Forty pairs of names, words, and non-words were selected for study. Additional names, words, and non-words were selected as practice stimuli. Target and lure lists were matched on mean length and number of syllables (and HAL frequency for word stimuli). Male and female names were paired in all possible combinations: female–female, male–male, male–female and female–male. Names were paired to avoid possible pre-existing commonly known associations (e.g., “Angela-Brad” which might be easy to associate with Angelina Jolie and Brad Pitt). Words were paired so as to avoid integrative [15] and common associations (e.g., “baby-diaper”). For the experimental stimuli, the 60 pairs of names, 60 pairs of words, and 60 pairs of non-words were separated into three lists of 30 pairs of names, 30 pairs of words, and 30 pairs of non-words. Lists were matched on measures of length and frequency, within stimulus type.

Participants were assigned randomly to one of the three stimulus type conditions (words, names, non-words). Order of test type (item or associative) was counterbalanced across participants. Test order (item then associative, associative then item) was counterbalanced across two blocks. The study phase, and item and associative recognition tests were presented on slides in a group setting. Participants completed the Salthouse [58] pattern comparison task as distractor task between the study and recognition test portions of the two blocks.

Two questionnaires were developed to measure memory self-efficacy (MSE) for item (MSEQ-I) and associative (MSEQ-A) recognition test abilities [59]. The questionnaires asked participants to judge their ability to recognize items and pairs of items. The general instructions on both questionnaires were “Circle YES if you believe you could do the task, and circle a percent confidence rating for how sure you are that you could do the task. Circle NO if you believe you cannot do the task (and do not circle a confidence rating).” The MSEQ-I is comprised of eight items of increasing levels of task difficulty ranging from lowest “If I studied 30 name-pairs for 5 seconds each, I could later identify 1 to 5 of the names correctly from a set of 40 items” to highest “If I studied 30 name-pairs for 5 seconds each, I could later identify 36 to 40 of the names correctly from a set of 40 items.” Participants made “Yes” or “No” responses at each level, and for each “Yes” response they rated how confident they were that they would achieve that level of performance by circling a confidence value from 10 to 100% (in 10% increments).

The MSEQ-A instructions were virtually identical to those of the MSEQ-I but differed in specific wording and number of items, reflecting the inherent structural differences between the item and associative recognition memory tests. Specifically, there were half the number of items on the MSEQ-A than on the MSEQ-I because the associative test is comprised of 20 pairs (of 40 items) and the item test is comprised of 40 items. As on the MSEQ-I, items on the MSEQ-A increased in difficulty, ranging from lowest “If I studied 30 word-pairs for 5 seconds each, I could later identify 1 to 5 of the pairs correctly from a set of 20 word-pairs” to highest “If I studied 30 word-pairs for 5 seconds each, I could later identify 16 to 20 of the words correctly from a set of 20 word-pairs.” Participants responded “Yes” or “No” at each level and for each “Yes” response also rated how confident they were that they would achieve that level of performance by circling a confidence value from 10 to 100% (in 10% increments). MSE scores were calculated by averaging confidence ratings across all levels (with “No” responses scored as zero), resulting in two summary scores, one each for MSEQ-I and MSEQ-A.

A questionnaire was developed to assess participants’ post-test perceptions of perceived task success, strategy use, task difficulty, effort expenditure, fatigue (“tiring”), and stamina (“keeping up with the task”) levels (see S1 *Questionnaire*). First, participants in each condition (words, names, nonwords) were asked to rate how much they had remembered on the item test and on the associate test. This is henceforth referred to as participants’ postdictions of their memory performance. Participants rated their memory for each test type by making a mark on a line ranging from 0% to 100% where 0% meant “not remembering anything” and 100% meant “remembering everything.” These marks were then transformed to percentages for the postdiction measure. Second, participants were asked if they had used any “tricks” or strategies to help learn the words and the pairings for the names, words, and non-words. These strategies were not coded for analysis but rather were used to prompt participants subsequently for their perceptions of how successfully they had used the strategies during the recognition tasks. Specifically, participants were asked, “What proportion of the time do you estimate that you successfully used the above strategies?” Participants rated perceived strategy success on a scale from 0% (“my strategies were not successful”) to 100% (“my strategies were always successful”). These estimates were recorded for each task and were used in analyses as a measure of perceived strategy success. Additionally, participants rated the amount of task difficulty, task effort, fatigue, and how easy it was to “keep up” for each task (“stamina”) on scales ranging from 1 (not at all) to 5 (very much).

### Procedure

Participants were tested in group sessions ranging from 2–10 participants per session. When they arrived for their session, they received a packet of materials, including consent form and testing materials, and were seated in classrooms at individual desks. The study was introduced to participants, and informed consent was obtained and demographic data were collected. The experiment was conducted using Powerpoint software. Instructions and tests were presented on a screen at the front of the room on a projector. The experimenter read aloud instructions projected on-screen regarding the item and associative tests. Participants then completed two short practice blocks. In the practice study phase, six pairs of items were presented one at a time for study. Participants were instructed to try to memorize the items and pairs for the upcoming tests. Each pair was presented on the screen for 5 seconds. A fixation point (+) appeared during a 1-sec interstimulus interval. After the practice study phase, participants completed sample items from the pattern comparison task [58] as a distractor task. Participants then completed practice item and associative recognition tests. In the practice item recognition tests, participants were presented with eight single names, words, or non-words. Half the items had been studied and half were new items that had not been studied. Participants recorded their recognition responses on numbered answer sheets. They were instructed to circle “yes” if they recognized the item as one which had been studied and “no” if they did not. In the practice associative recognition tests, participants were presented with four pairs of words, names, or non-words. Half of these pairs were presented intact (i.e., the original pairings from the study phase) and half were presented in recombined pairs. In the recombined pairs, both items in the pair had been presented in the study phase but had been paired with different items. The task in the associative test was to circle “yes” if the pair was an intact pair from the study phase and “no” if the pair was a recombined pair. The order of practice tasks (item/associate) was counterbalanced across participants.

After the practice trials, participants completed the MSEQ-I and MSEQ-A to assess memory self-efficacy for the recognition tasks before they began the experimental blocks. Half the participants completed the MSEQ-I first and half completed the MSEQ-A first. Next, for the study phase, 30 stimulus pairs were presented on a projector at the rate of 5 sec per pair with a 1-sec interstimulus interval during which a fixation cross was shown. After study, participants completed the pattern comparison distractor task and were given 20 seconds to match as many patterns as possible. Participants then completed the item and associative recognition tests (order counterbalanced). The procedures for the item and associative tests were identical to those in the practice blocks. In the item tests, 40 items (words, names, or non-words) were presented, 20 of which were previously studied and 20 of which were new. In the associative tests, 20 name-pairs, word-pairs, or non-word-pairs were presented; 10 of the pairs were intact and 10 of the pairs were recombined.

## Results

### Testing the associative deficit hypothesis

**Fig 1:**
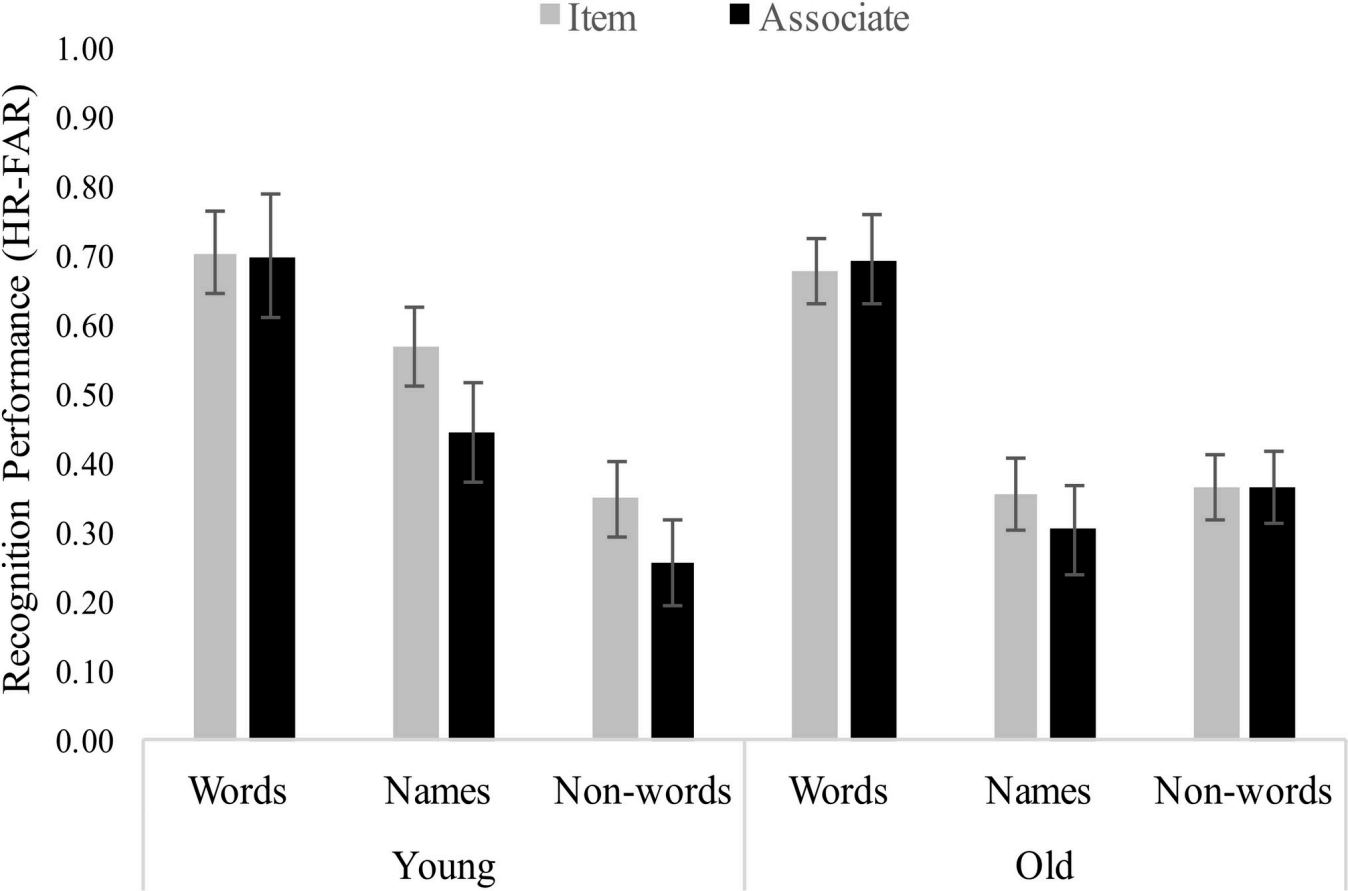
Recognition performance (hit rate minus false alarm rate) by age, stimulus type, and test type.

Recognition memory scores (hit rate minus false alarm rate) were entered into a mixed analysis of variance (ANOVA) with Age (young, old) and Stimulus Type (words, names, nonwords) as between-participants factors and Test Type (item, associate) as a within-participants factor. The main effect of Stimulus Type was significant, *F* (2,100) = 26.90, *p* < .001, *n*_*p*_^*2*^ = .35, with average recognition performance highest for words (*M =* 0.69, *SD* = 0.23), intermediate for names (*M* = 0.43, *SD* = 0.24), and lowest for nonwords (*M* = 0.33, *SD* = 0.18) (see Fig 1). Post-hoc analyses revealed a significant difference between recognition for words and names, *t*(142) = 5.69, *p* < .001, Cohen’s *d* = 0.949 words and nonwords, *t*(138) = 8.39, *p* < .001, Cohen’s *d* = 1.422 and a marginal difference between recognition for names and nonwords, *t*(138) = 2.24, *p* = .027, Cohen’s *d* = 0.379. These post-hoc comparisons were interpreted with Bonferroni corrections (for 3 tests; *p* < .0167). Cohen’s *d* does not correct for multiple comparisons. For all ANOVA results, post-hoc comparisons, as well as analyses with hit rates and false alarm rates, see S1 *Appendix*.

No other main effects and interaction effects were significant. Notably, older and younger adults had similar recognition memory scores overall (50% for younger adults and 47% for older adults). Further, the lack of a significant main effect for Test Type provides no support for an associative deficit, and the nonsignificant Age by Test Type interaction effect is inconsistent with the associative deficit hypothesis [4]. However, in a supplemental analysis, false alarm rate was significantly greater for the associative task than item task, overall. No significant effects of age on false alarm rate or hit rate were found (see S1 *Appendix*). Possible explanations for the recognition results include procedural differences compared to other studies of the associative deficit. These issues are examined further in the *Discussion*.

### Age-related differences in metacognition

In order to provide statistical comparisons between older and younger adults’ metacognitive data, we conducted two-tailed *t*-tests between younger and older adults on each of the eight metacognitive variables for item and associative tests. Table 2 shows average recognition performance scores and rating scores on the metacognitive variables by Test Type and Age with the results of the age comparisons for each Test Type.

**Table 2:** Performance and metacognition means and standard deviations by age and test type.

Variable	Younger Adults (N = 57)		Older Adults (N = 49)		t-test p _bonf_ values and Cohen’s d	
	Item	Associate	Item	Associate	Item YA vs. OA	Associate YA vs. OA
Performance (HR—FAR)	0.54 (0.29)	0.46 (0.37)	0.48 (0.25)	0.47 (0.30)	*p =* 1	*p =* 1
MSEQ (1–100)	49.50 (18.45)	59.69 (18.46)	38.49 (23.11)	48.37 (22.94)	*p =* .136	*p =* .105
Postdictions (1–100)	58.26 (19.08)	57.28 (25.87)	57.12 (21.69)	51.12 (23.53)	*p =* 1	*p =* 1
Postdiction Accuracy	-0.19 (0.17)	-0.16 (0.19)	-0.17 (0.17)	-0.22 (0.21)	*p =* 1	*p =* 1
Strategy (1–100)	56.14 (23.60)	57.32 (25.90)	46.18 (25.59)	41.57 (27.63)	*p =* .715	*p =* .056
Difficulty (1–5)	3.53 (1.00)	3.51 (1.10)	3.69 (1.10)	4.04 (1.14)	*p =* 1	*p =* .294
Effort (1–5)	3.68 (0.74)	3.86 (0.79)	4.02 (0.97)	4.18 (0.88)	*p =* .812	*p =* .874
Fatigue (1–5)	**2.79 (1.13)**	2.75 (1.14)	**1.88 (1.09)**	2.06 (1.21)	***p* < .001, *d* = .82**	*p* = .055
Stamina (1–5)	1.33 (0.61)	1.33 (0.61)	1.22 (0.55)	1.39 (0.84)	*p* = 1	*p* = 1

*Note*. Standard deviation values are shown in parentheses. Two-tailed *t*-tests were conducted between younger and older adults on each variable for item and associative tests separately. Results with Bonferroni corrections (for 18 tests; *p* < .0028) are reported in the fifth and sixth column. Cohen’s *d* does not correct for multiple comparisons.

MSEQ indicates average ratings from the Memory Self-Efficacy Questionnaire.

Postdiction Accuracy = Postdiction Rating minus Percentage Correct. Zero indicates a perfect match between postdictions and performance accuracy. A negative value indicates underestimation and a positive value indicates overestimation of performance.

The only statistically significant difference in age was for fatigue on the item test, *t*(104) = 4.207, Bonferroni-corrected *p* < .001, uncorrected Cohen’s *d* = 0.820. Younger adults reported significantly more fatigue (*M =* 2.79, *SD* = 1.13) than older adults (*M* = 1.88, *SD* = 1.09) on the item test. However, no other significant age differences for performance or metacognition were found using univariate statistics. These results motivated us to conduct exploratory multivariate analyses using neural networks. The data for variables summarized in Table 2 were subjected to neural network analyses, reported below.

### Individual differences in older adults’ metacognition

We performed an exploratory analysis of individual differences with artificial neural networks trained to categorize individuals as young or old using the entire variable space (i.e., recognition scores, postdictions, strategies, etc.). Our reasoning behind this approach was that if the network was able to adequately categorize participants as young or old using the data as input, probing the network’s internal structure would show useful information about the differences in memory and metacognition used to make this categorization between young and old. That is, the network would reveal multivariate patterns distinguishing younger adults from older adults.

Artificial neural networks are powerful tools for discriminating between individuals (e.g., for review, see [53, 60–63] and have been used in other fields for this purpose. For example, Wisniewski et al. [63] found that artificial neural networks revealed individual differences in learning/memory strategies that predicted later performance on a perceptual memory task. Cohen [64] trained an artificial neural network to discriminate between children with autism and children with mental retardation, based on information obtained during interviews with caregivers. Recently, researchers have begun using artificial neural networks to study the effects of treatment in children with autism as an alternative to observational methods, which have been shown to have poor internal validity [65]. Thus, we sought to apply the power of neural network analyses to examine patterns of metacognitive processing differences in younger and older adults within the context of the associative memory deficit.

**Fig 2:**
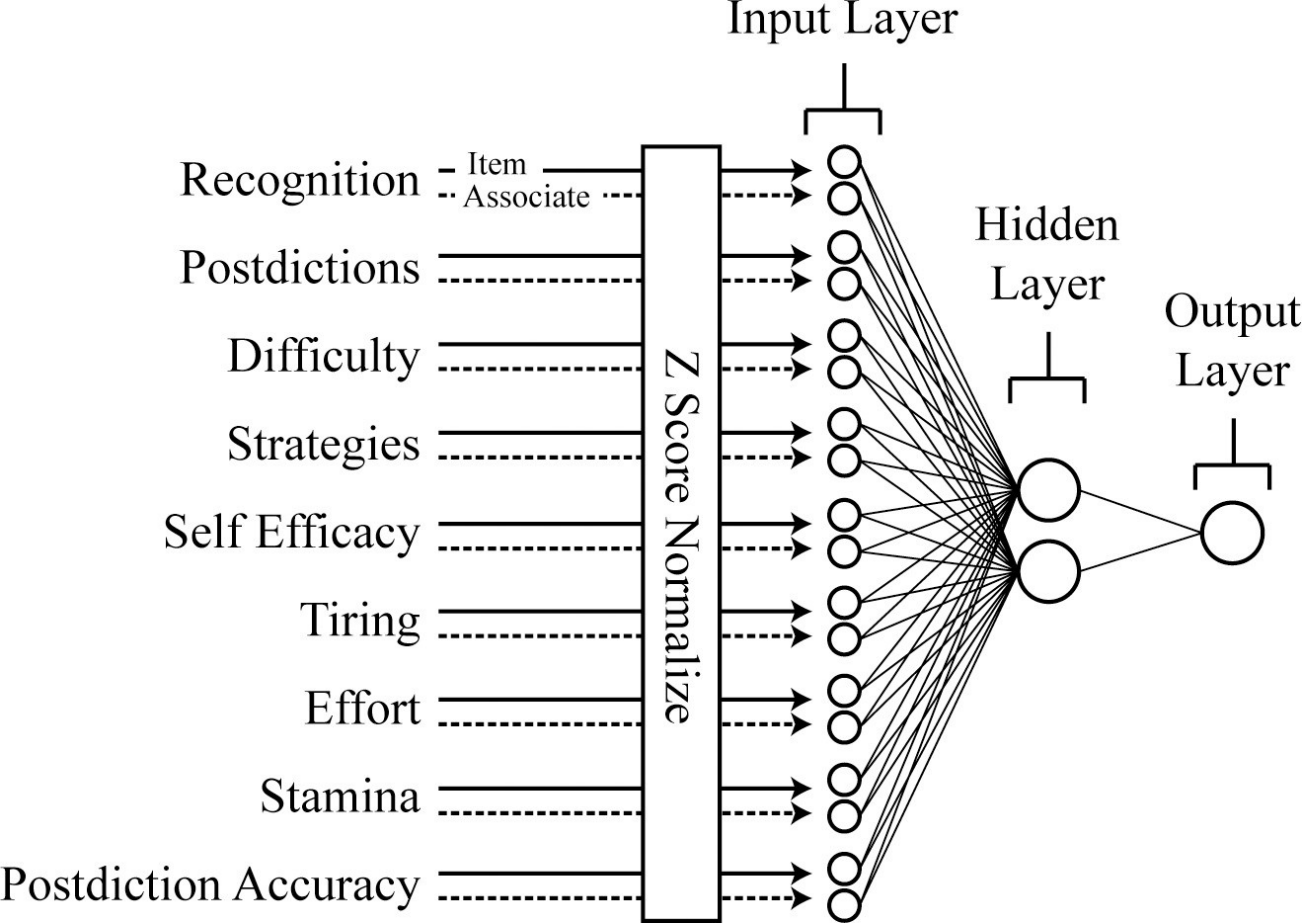
Architecture of the model employed. Recognition performance and the seven metacognitive variables were Z-score normalized and these scores were used as input activations in the input layer of the model. The hidden layer contained two units, and the output layer was made up of a single output unit. The network was trained to respond with an activation of 1 in the output unit when presented with an older adult’s data and -1 if presented with a younger adult’s data.

All artificial neural network procedures were conducted in Matlab R2015b using the neural network toolbox for Matlab and custom scripts and functions. A depiction of the employed neural network model is given in Fig 2. The experimental dependent variables were first Z-score normalized. These Z-scores were used as input activations for 18 units in the input layer. The input layer was fully connected via weighted connections to 2 hidden layer units, and then to a single output unit. All units employed logistic activation functions, squashing the sum of weighted input plus a bias parameter, to values between -1 and 1. As is typical with such a neural network architecture, when an input (here, data corresponding to a single individual) was presented to the network, that input led to activation in the 2 hidden units based on those units’ weighted connections to the input layer and their biases. Hidden unit activation then led to activation in the single output unit based on the output unit’s weighted connections to the hidden units and the output unit’s bias.

Weights and biases in the network were initially set to random values between -.005 and .005. A target output activation was associated with each individual such that it was equal to -1 for younger adults and +1 for older adults. A network with adequate weights and biases would therefore be able to produce an activation near +1 in the output unit when presented with an older participant’s data and an activation of -1 when presented with a younger participant’s data. However, before any training, networks were expected to have less than adequate weights and biases to make such a classification. Through training from iterations of error-correction, the weights and biases were gradually adapted to make the classification (for a review, see [53]). See S2 *Appendix* for more details on the parameters and learning rules for the employed artificial neural network. A supplementary analysis was conducted to test whether results obtained with the employed neural network were replicable and robust to changes in network architecture. The trends reported in this paper were replicated in 20 additional runs of retraining the model and with a model containing 3 hidden units. See S3 *Appendix* for figures that report this analysis.

The trained network was able to accurately categorize virtually the entire sample. Of the 106 participants, approximately 98% were categorized correctly (i.e., positive values for older adult, negative values for younger adult). One older adult was mistakenly categorized as young and one young adult was mistakenly categorized as old. We further tested network accuracy with a leave-one-out cross validation procedure. This let us quantify the degree to which a trained network of the selected architecture could classify individuals it had not been trained to classify. For 106 runs, one individual was left out of the inputs used for training, then tested after training. The signal detection parameter *d’* was 1.17 for classification of untrained individuals. This level of accuracy allowed us to probe the internal structure of the network trained on all individuals to determine how it was able to perform the young adult versus older adult classification.

**Fig 3:**
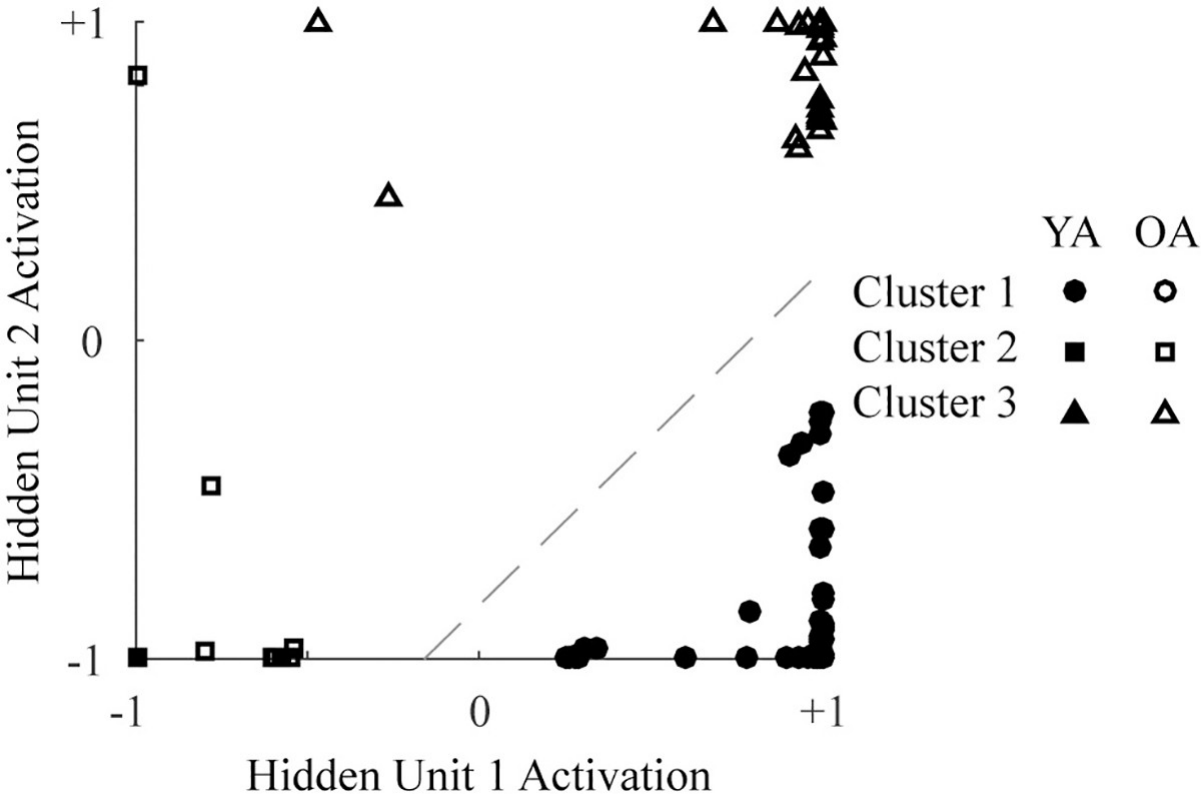
Activations within the hidden layer to each individual’s input data. The dashed line shows the decision boundary of the output unit. Filled markers represent younger adults (YA). Open markers represent older adults (OA). Marker shape (circle, square, or triangle) indicates cluster assignment as determined by k-means.

Fig 3 shows activations within the hidden unit layer to each individual’s input data with hidden unit 1 activation on the x-axis and hidden unit 2 activation on the y-axis. Filled markers in the figure represent young adults and open markers represent older adults. The dashed line represents the decision boundary of the single output unit of the network. The network grouped most younger adults in the lower right corner of this space (high activation of hidden unit 1 and low activation of hidden unit 2). Appropriately, the decision boundary of the output unit (dashed line) separated this part of hidden unit space from the rest, allowing low output activation to younger adults, but high output activation to older adults. Of note, and unlike younger adults, the older adults appeared to group into 2 different areas of the hidden unit space. One group of older adults showed high activation in both hidden units (upper right corner), whereas the other showed low activation in both hidden units (lower left corner). It thus appears that the network was able to successfully categorize younger adults and older adults by forming two distinct groupings of older adults and one grouping of younger adults.

An automated k-means procedure was used to blindly group individuals into 3 different clusters based on the hidden unit activations shown in Fig 3 (for review, see [53]). Determined cluster assignments (Cluster 1, Cluster 2, or Cluster 3) are represented in Fig 3 by the shape of markers. Cluster 1 (circles) contained 56 younger adults and 1 older adult in the lower right portion of the hidden unit space. Cluster 2 (squares) contained 1 younger adult and 12 older adults portioned out towards the lower left portion of the hidden unit space. Cluster 3 (triangles) contained 0 younger adults and 36 older adults located mostly in the upper right portion of hidden unit space. In general, this blind clustering aligned with our visual interpretation of the data (i.e., distinct groups of older adults identified by network hidden units).

**Fig 4:**
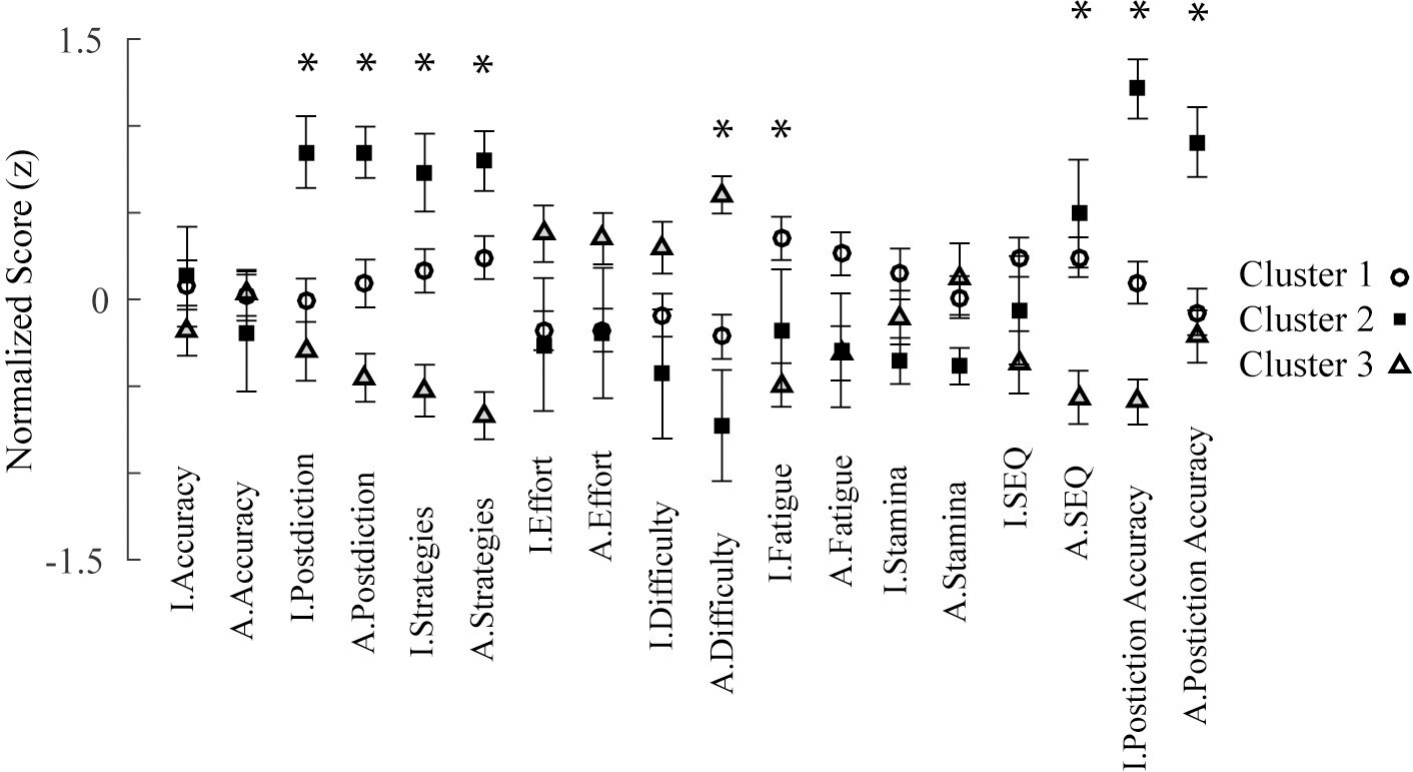
Mean normalized scores on each variable used as input for the network. Error bars show standard errors of the mean. Asterisks mark inputs showing significant differences between clusters (Bonferroni corrected). Partial eta squared effect sizes for significant effects in the neural network clusters are (left to right): .88, .91, .92, .95, .93, .90, .91, .96, .88.

To determine what features of the data related to these three different clusters, we looked at the means of the inputs within each cluster (Fig 4). In the figure, values above zero reflect a higher than average score on the associated dependent variables. Values lower than 0 reflect a lower than average score on the dependent variables. Many dependent variables showed little if any difference between the three clusters. For instance, item and associate accuracy were similar across clusters. This is consistent with the recognition performance ANOVA results (see Fig 2). Other dependent variables appeared to show large differences between clusters. To narrow down which features were most important for the network to form these clusters, one-way independent measures ANOVAs comparing the 3 different clusters were run on each of the dependent variables. These ANOVAs were interpreted with Bonferroni corrections (for 18 tests). Asterisks in the figure mark dependent variables showing significance (*p* < .0028).

Several dependent variables showed significant differences between clusters. Both item and associate postdictions showed high scores for Cluster 2 compared to Cluster 1 and Cluster 3, with Cluster 3 showing the lowest scores out of the 3. Cluster 2, which was made up of mostly older adults, showed relatively high ratings of their memory performance. In contrast, Cluster 3 older adults gave relatively low ratings of memory performance. A similar pattern was observable for item and associate strategies. That is, Cluster 2 participants reported higher perceived strategy-use success, Cluster 1 reported less perceived strategy-use success, and Cluster 3 reported least strategy-use success. On ratings of associate difficulty, Cluster 3 older adults gave relatively high ratings of difficulty, whereas Cluster 2 and Cluster 1 reported less perceived difficulty. For fatigue on the item task, younger adults (Cluster 1) gave relatively high ratings compared to the two clusters of older adults, consistent with results from inferential statistics between young and old (Table 2). For self-efficacy on the associate task, Cluster 1 younger adults and Cluster 2 older adults showed relatively high self-efficacy relative to Cluster 3 older adults, who showed lower self-efficacy. Finally, for both item and associate postdiction accuracy, Cluster 2 older adults showed highest postdiction accuracy compared to the younger adults making up most of Cluster 1 and the older adults making up all of Cluster 3.

The differences between clusters on the dependent variables reveal two important aspects of the data that distinguish clusters. First, differences exist strongly in the metacognitive measures, but not in the performance measures (i.e., recognition). Second, the two separate clusters made up of mostly older adults (Cluster 2 and Cluster 3) appear to represent opposite patterns in the data. Generally, Cluster 2 is a group of older individuals who find the tasks relatively easy, they believe they perform well, and these beliefs are accurate. In contrast, Cluster 3 older individuals show the opposite trend. They find the tasks difficult, they believe they are performing relatively poorly, and these beliefs do not map accurately to their own performance. Cluster 1 individuals, made up mostly of younger adults, fall somewhere in between Cluster 2 and Cluster 3, with the exception of ratings of fatigue (cf. ANOVA results above).

An unexpected, but interesting component of the patterns that distinguished clusters, was that there were dependent variables from the associative task that distinguished clusters, whereas the corresponding dependent variables from the item task were not as informative. For instance, both associative difficulty ratings and associative self-efficacy were informative, especially for differences between the two clusters made up of mostly older adults (Cluster 2 and Cluster 3). Similar to Berry et al. [2], the metacognitive measures of associative memory reveal greater between age group differences than the item measures. To explore this possibility a separate set of network simulations was conducted. Instead of training on the entire set of dependent variables, new networks were trained with either dependent variables corresponding to the item or dependent variables corresponding to the associative task. A leave-one-out cross-validation procedure ([66, 67]; for review, see [68]) was used to test how well networks were able to categorize younger adults and older adults using either inputs corresponding to the item or associative task. In this procedure, a network was initialized and trained using the methods described above. However, instead of being trained with data from each individual, a network was trained with every individual except for one. This procedure was repeated 106 times so as to train a network while leaving out each individual in the dataset. Networks were then tested for whether or not they were able to correctly classify the left out individual as a younger adult or older adult. The signal detection measure *d’* was used as the measure of performance. Networks trained with data corresponding to the item task had a *d’* = 0.77. Networks trained with data corresponding to the associative task had a *d’* of 1.43. Thus, data from the associative task were more useful for making the younger versus older categorization than data from the item task.

## Discussion

Neural network modeling supported a dissociation of younger from older adults using metacognitive and memory measures. Artificial neural network analyses discovered two distinct groups of older adults. Specifically, high-performing older adults (Cluster 2) appear to possess distinct, metacognitive qualities as measured by perceived strategy success, postdictions, and postdiction accuracy, that are not shared by other older adults (Cluster 3) and younger adults (Cluster 1). These results highlight the importance of considering individual differences in tests of associative memory deficits, especially within older adults. Most studies collapse across older adults, treating them as one group. Using artificial neural networks, the present study discovered two discrete groups of older adults who monitored their performance in different ways and levels of accuracy. This is a novel finding in the memory and metacognitive aging literature and has implications for the associative deficit hypothesis.

Initially, this study tested the associative deficit hypothesis [4] using three stimulus conditions: words, names, nonwords. We expected to find an age-specific associative deficit for words but not for names and nonwords. None was found in any stimulus condition. In fact, older and younger adults performed comparably across all stimulus conditions. Previous work also failed to find an age-specific associative deficit using non-words [1] and names [2]. However, the lack of support for an associative deficit using word stimuli is inconsistent with much of the literature (e.g., [2, 4, 49, 50, 29–31]). There are several possible explanations for the absence of the associative deficit in the present study, which will be discussed later.

The primary goal of this study was to examine age and individual differences in memory and metacognition. In addition to recognition performance, we collected seven metacognitive measures: difficulty, perceived strategy success, self-efficacy, fatigue, effort, stamina, and postdictions. We then also calculated an eighth metacognitive variable, postdiction accuracy, by subtracting each participant’s percentage correct by her or his postdiction rating (*How much did you remember in this study*?) in item and associative tasks. We entered these data, along with individual’s recognition performance, into an artificial neural network that was trained to differentiate between older and younger adults. Using two sets (item and associative) of nine dependent variables, three distinct groups (clusters) of participants were found.

Cluster 1 comprised all but one young adult in the study. Cluster 2 was made up of twelve older adults and one young adult. The remaining older adults belonged to Cluster 3. All three groups performed about the same on item and associate tests. What made them different were their metacognitive ratings. The variables that were significantly different between groups were: postdictions, perceived strategy success, difficulty (associate only), fatigue (item only), self-efficacy (associate only), and postdiction accuracy (see Fig 4).

A crucial finding was that Cluster 2 and Cluster 3, both of which contained mostly older adults, looked very different on these measures. Cluster 2 found the tasks relatively easy, they believed they performed well (high postdictions), and these beliefs were accurate (high postdiction accuracy). In contrast, Cluster 3 individuals found the tasks to be difficult, they believed they were performing relatively poorly (low postdictions), yet these beliefs did not map accurately onto their own performance (low postdiction accuracy). Cluster 1 individuals, made up mostly of young adults, fell somewhere in between Cluster 2 and Cluster 3, with the exception of ratings of fatigue. Interestingly, Cluster 2 had both high postdiction accuracy and high perceived strategy success while the remaining older adults (Cluster 3) had low postdiction accuracy and low perceived strategy success. That is, older adults who are better at judging their overall performance accuracy are also more confident in their strategy effectiveness. This pattern supports Hertzog and Dunlosky’s [11] framework, which states performance monitoring provides information that can be used to draw inferences about strategy effectiveness ([11], p. 222). Research exploring this relationship between strategy use and metacognition in older adults is growing (e.g., [33, 69–72]).

Next, we asked which set of task measures (item or associate) was better at discriminating between three clusters of participants. Instead of training on the entire set of dependent variables, new networks were trained with either dependent variables corresponding to the item task or dependent variables corresponding to the associative task. We found that networks trained with data corresponding to the associative task had a *d’* that was 0.66 greater than networks trained with data corresponding to the item task. Thus, data from the associative task were more useful for discriminating younger vs. older adults than data from the item task. This finding is consistent with much of the associative deficit literature. While older and younger adults typically perform about the same on the item test, performance on the associative test is what distinguishes younger and older participants (e.g., [4]). Although we did not find this difference between younger and older adults in associative performance, neural network modeling found differences between younger and older adults in associative metacognition. That is, while associative memory looked the same for older and younger participants, patterns of metacognitive ratings for the associative task looked different. This result highlights the importance of evaluating metacognition in associative memory paradigms. It would be interesting to test whether similar differences based on metacognitive data are revealed in previous and future associative memory studies, including those using cued- and free-recall test paradigms, each of which tend to require more self-initiated associative retrieval processes than recognition test paradigms [73, 74]. Recently, McGillivray and Castel [33] found both younger and older adults displayed equivalent metacognitive abilities in cued and free recall tasks that incentivized memory performance with a point system.

Why did we fail to find an age-specific associative deficit? The age-specific associative deficit has been partially attributed to older adults’ tendency to say they remember recombined word pairs [2, 3, 75]. It is reasonable why this error is common. The associative task is especially challenging because any sense of familiarity to the studied word must be overcome in order to correctly “reject” a recombined word pair. The associative task, therefore, relies on recollective processes, such as explicit retrieval of mediators used during encoding. For example, one might recollect a mental image of a wardrobe sinking to the bottom of the ocean for *wardrobe-ocean*. Alternatively, one might retrieve the sentence they generated to include both words in the word pair (e.g., “My wardrobe is filled with clothing to wear at the ocean”). Older adults have a particularly hard time with this task, ultimately, producing more errors than younger adults. One reason for this is because yes/no recognition tests place high demands on self-initiated retrieval strategies [24]. Our failure to find an associative deficit may be the result of task procedures and testing that could have provided support during retrieval in the following ways.

First, participants were provided with information on the number of test trials for items and associates during the instructions phase. That is, participants were told equal numbers of intact and recombined pairs (as well as old and new items) would be presented at test. During testing, participants made “yes/no” responses on a numbered response sheet that was completed by hand. So, as participants completed their response sheet, they may have kept track of how many “yes” and “no” responses they have made in each task. This additional structure may have led older adults in our study to respond more conservatively, by distributing their “yes” responses during the associative task more equivalently across the two types of test pairs, thereby decreasing false alarm rates and enhancing overall memory accuracy in older adults. In fact, in a supplemental analysis, we found no significant effect of age on false alarm rate (see S1 *Appendix*). This explanation is consistent with Craik’s [76] argument that environmental support at retrieval reduces older adults’ memory deficits (also, see [77]). In cued recall tasks, for example, older adults’ performance is comparable to younger adults’ performance when four letters (versus three letters) are given in the stem at retrieval, suggesting that younger and older adults’ memory processes are more similar when increased support is provided at retrieval [78].

Second, our study was also administered in a group setting, with approximately 5–6 participants at a time. Group testing might have provided social support that we did not expect, especially for older participants. While young adults are used to being tested during in-class exams and quizzes (they are current college students), older adults may experience anxiety about being tested on their memory. After all, many older adults have already noticed some memory deficits in their everyday lives. For older adults, a group setting might have provided extra social support from their peers. This could have reduced anxiety and allowed for increased performance on the associative task, on which older adults normally struggle.

Finally, it should be noted that participants completed memory self-efficacy questionnaires prior to the study and memory test phases, which may have inadvertently created a negative priming context for older adults. Research on stereotype threat shows that older adults who are primed with negative information about memory and aging prior to a memory test perform more poorly than younger adults on the memory test [79], and more poorly that older adults who receive neutral or positive primes [80]. Yet, in our study, performance on the item and associate memory tests did not differ significantly between the older and younger adults. These results render unlikely the possible operation of stereotype threat in our study but future research might want to systematically assess the impact of self-efficacy ratings collected prior to versus after the item and memory tests in the associative deficit paradigm.

An important outcome from this failure to produce the age-specific associative deficit was the exploration and discovery of individual differences in metacognition. Currently, the literature on metacognition and aging has produced mixed results. While some have suggested that age-related associative deficits may be related to poor metacognitive monitoring (e.g., [14]), others have suggested that metacognitive accuracy (how well one’s metacognitive judgments track his or her performance accuracy) may not necessarily decrease with age (e.g., [39, 81–85]). For example, while older adults have shown impaired memory performance compared to younger adults, they still made accurate estimations about forgetting [83]. Additionally, because memory recognition and the associative deficit vary by task type (e.g., [8, 24]) and stimulus type [1, 2], it is not surprising that patterns in metacognitive responding vary between studies. Our findings suggest that a subset of older adults (here, approximately 26% of older adults) with high levels of performance and exceptional metacognitive monitoring skills might influence overall trends in memory, metacognition, and aging results.

Inferential statistics comparing younger and older participants’ performance and eight metacognitive measures on item and associative tasks yielded just one significant result: younger adults showed greater fatigue than older adults on the item test (Table 2). However, when we entered these data into a neural network, we discovered important differences between a subset of older adults that would have otherwise gone undetected. Perhaps, the most compelling finding from this multivariate analysis was that data from the associative task were more useful for neural networks to discriminate between younger and older adults than data from the item task. This has important implications for the associative memory and metacognitive aging literature. While older and younger adults did not vary on the associative recognition memory test, their patterns of metacognitive ratings for the associative test were different. Only through use of a relatively novel statistical tool in aging research—artificial neural network modeling—did we discover the importance of metacognitive measures in associative memory. Our analysis differentiated between younger and older adults as well as identified individual differences within this group of older adults. We encourage others to explore the individual differences in their own data. Artificial neural network modeling is a useful data-mining tool for this purpose.

## Supporting information

S1 Appendix ANOVA results, post hoc comparisons, and additional ANOVAs. Includes recognition ANOVA results reported in the paper with post-hoc comparisons as well as two additional ANOVAs with 1) hit rate and 2) false alarm rate as dependent variables.
(DOCX)

S2 Appendix Artificial neural network parameters. (DOCX)

S3 Appendix Supplemental artificial neural network analysis. (DOCX)

S1 Questionnaire Post-test questionnaire. Questionnaire used to collect postdictions, strategies, effort, difficulty, tiring (“fatigue”), and keeping up (“stamina”) in the “words” stimulus condition. Post-test questionnaire for names and nonwords were identical except for change in stimulus term (“word”, “name”, “nonword”).
(DOCX)

S1 Dataset Raw data used for all analyses. (CSV)
