# Correction: Artificial neural networks reveal individual differences in metacognitive monitoring of memory

**DOI:** 10.1371/journal.pone.0222644

**Published:** 2019-09-12

**Authors:** Alexandria C. Zakrzewski, Matthew G. Wisniewski, Helen L. Williams, Jane M. Berry

The following information is missing from the Funding statement: Publication of this article was funded in part by the Kansas State University Open Access Publishing Fund.
