# Supplementary material for: Artificial neural networks reveal individual differences in metacognitive monitoring of memory
Source: PLoS One. 2019 Jul 31;14(7):e0220526. doi: 10.1371/journal.pone.0220526 (PMC6668824; doi:10.1371/journal.pone.0220526)
Supplement: S1 Appendix — Includes recognition ANOVA results reported in the paper with post-hoc comparisons as well as two additional ANOVAs with 1) hit rate and 2) false alarm rate as dependent variables. (DOCX) [file pone.0220526.s001.docx]

**S1. Appendix**

Below are the results of the test for the associative deficit hypothesis with recognition memory scores (hit rate minus false alarm rate) entered into a mixed analysis of variance (ANOVA) with Age (young, old) and Stimulus Type (words, names, nonwords) as between-participants factors and Test Type (item, associate) as a within-participants factor. Post-hoc two-tailed *t*-tests examining comparisons of interest (Test Type, Age, Stimulus Type, and Age for each Stimulus Type by Test Type) were conducted with Bonferroni corrections. Main effect of Stimulus Type as well as Stimulus Type comparisons are reported in the main text (see section *Testing the associative deficit hypothesis*).

**Repeated Measures ANOVA**

**Within Subjects Effects**

|  | **Sum of Squares** | **df** | **Mean Square** | ***F*** | ***p*** | **η² _p_** |
| --- | --- | --- | --- | --- | --- | --- |
| Test Type | 0.096 | 1 | 0.096 | 2.102 | 0.150 | 0.021 |
| Test Type x Age | 0.052 | 1 | 0.052 | 1.150 | 0.286 | 0.011 |
| Test Type x Stimulus | 0.078 | 2 | 0.039 | 0.855 | 0.428 | 0.017 |
| Test Type x Age x Stimulus | 0.012 | 2 | 0.006 | 0.128 | 0.880 | 0.003 |
| Residual | 4.547 | 100 | 0.045 |  |  |  |

**Between Subjects Effects**

|  | **Sum of Squares** | **df** | **Mean Square** | ***F*** | ***p*** | **η² _p_** |
| --- | --- | --- | --- | --- | --- | --- |
| Age | 0.099 | 1 | 0.099 | 1.071 | 0.303 | 0.011 |
| Stimulus | 4.967 | 2 | 2.483 | 26.900 | < .001 | 0.350 |
| Age x Stimulus | 0.515 | 2 | 0.258 | 2.791 | 0.066 | 0.053 |
| Residual | 9.232 | 100 | 0.092 |  |  |  |

*Note.*  Type III Sum of Squares

**Post Hoc Comparisons**

**Test Type**

|  | **Mean Difference** | ***t*** | **df** | **Cohen’s *d*** | ***p* _bonf_** |
| --- | --- | --- | --- | --- | --- |
| Item vs. Associative | 0.046 | 1.57 | 211 | 0.153 | 1 |

**Age**

|  | **Mean Difference** | ***t*** | **df** | **Cohen’s *d*** | ***p* _bonf_** |
| --- | --- | --- | --- | --- | --- |
| Younger vs. Older | 0.029 | 0.686 | 210 | 0.095 | 1 |

**Stimulus Type**

|  | **Mean Difference** | ***t*** | **df** | **Cohen’s *d*** | ***p* _bonf_** |
| --- | --- | --- | --- | --- | --- |
| Words vs. Names | 0.265 | 5.69 | 142 | 0.949 | 0.000 |
| Words vs. Nonwords | 0.363 | 8.39 | 138 | 1.422 | 0.000 |
| Names vs. Nonwords | 0.098 | 2.24 | 138 | 0.379 | 0.297 |

**Age by Stimulus and Test Type**

|  | **Mean Difference** | ***t*** | **df** | **Cohen’s *d*** | ***p* _bonf_** |
| --- | --- | --- | --- | --- | --- |
| Item: Words: YA vs. OA | 0.028 | 0.36 | 34 | 0.120 | 1 |
| Item: Names: YA vs. OA | 0.214 | 2.73 | 34 | 0.982 | .108 |
| Item: Nonwords: YA vs. OA | 0.017 | 0.22 | 32 | 0.078 | 1 |
| Assoc.: Words: YA vs. OA | 0.006 | 0.05 | 34 | 0.017 | 1 |
| Assoc.: Names: YA vs. OA | 0.139 | 1.38 | 34 | 0.469 | 1 |
| Assoc.: Nonwords: YA vs. OA | 0.109 | 1.30 | 32 | 0.455 | 1 |

*Note.*  Two-tailed *t*-tests were conducted and interpreted with Bonferroni corrections (for 11 tests; *p* < .0045). Cohen’s *d* does not correct for multiple comparisons.

**ANOVA with hit rates**

In addition to testing the associate deficit hypothesis with recognition memory scores (described above), we also entered hit rates into the same mixed analysis of variance (ANOVA) with Age (young, old) and Stimulus Type (words, names, nonwords) as between-participants factors and Test Type (item, associate) as a within-participants factor. This analysis replicated our recognition (HR-FAR) findings, with a significant main effect of Stimulus Type, *F* (2,100) = 16.55, *p* < .001, *n_p_^2^* = 0.249. Mean hit rates for Stimulus Types followed the same pattern as in the recognition performance, with average hit rate highest for words (*M* = 0.84, *SD* = 0.16), intermediate for names (*M* = 0.72, *SD* = 0.19), and lowest for nonwords (*M* = 0.66, *SD* = 0.16). Again, we found no other significant main effects or interactions. Older and younger adults had similar hit rates overall (76% for younger adults and 72% for older adults).

**ANOVA with false alarm rates**

We also entered false alarm rates into the same mixed analysis of variance (ANOVA) with Age (young, old) and Stimulus Type (words, names, nonwords) as between-participants factors and Test Type (item, associate) as a within-participants factor. Once again, we found a significant main effect of Stimulus Type, *F* (2,100) = 16.18, *p* < .001, *n_p_^2^* = 0.244. Mean false alarm rates were lowest for words (*M* = 0.14, *SD* = 0.18), intermediate for names (*M* = 0.29, *SD* = 0.18), and highest for nonwords (*M* = 0.33, *SD* = 0.17), consistent with the trend shown for recognition and hit rate. Here, there was also a significant main effect of Test Type, *F* (1,100) = 16.70, *p* < .001, *n_p_^2^* = 0.143, such that false alarm rate was higher for the associative task (*M* = 0.29, *SD* = 0.20) than the item task (*M* = 0.22, *SD* = 0.17). The main effect of Test Type is not surprising, given that the associative task presents recombined words, with lures seen during the study phase, while the item task includes new words not seen before. Participants are more likely to false alarm to recombined words based on familiarity in the associative task compared to than to new items in the item task. See *Discussion* section for more characterizations of the associative task. No other significant main effects or interactions were found. Older and younger adults had very similar false alarm rates overall (26% for younger adults and 25% for older adults).
