# Supplementary material for: Artificial neural networks reveal individual differences in metacognitive monitoring of memory
Source: PLoS One. 2019 Jul 31;14(7):e0220526. doi: 10.1371/journal.pone.0220526 (PMC6668824; doi:10.1371/journal.pone.0220526)
Supplement: S2 Appendix — (DOCX) [file pone.0220526.s002.docx]

**S2. Appendix**

This appendix describes in further detail the parameters and learning rules for the employed artificial neural network. As detailed above, weights and biases in the network were initially set to random values between -.005 and .005. A target output (*t_j_*) was associated with each individual such that *t_j_* was equal to -1 for younger adults and +1 for older adults. Before any training, networks were expected to produce considerable error (*δ_j_*) as defined by equation 1.

$\delta_{j}=\left( t_{j}- a_{j} \right)a_{j}(1-a_{j})$ (1)

Here, the difference between *t_j_* and the network’s actual output unit activation (*a_j_*) is scaled by the derivative of the logistic activation function employed by units (*a_j_*(1- *a_j_*)). Key is that if the network’s actual output (*a_j_*) is far from the target output (*t_j_*), *δ_j_* is large. Networks were trained to categorize younger adults and older adults through error correction. In each training iteration, weights between the output unit and hidden unit *i* (Δ*w_ij_*) were changed according to equation 2, and the bias of the output unit (*b_j_*) was changed according to equation 3.

${\Delta w}_{ij}=\eta\delta_{j}a_{i}$ (2)

${\Delta b}_{j}=\eta\delta_{j}$ (3)

Here, *η* is a learning rate parameter set to .01 and *a_i_* is the activation of hidden unit *i*. The generalized delta rule was used to backpropogate error from the output unit to hidden units, changing connections between input units and hidden units in a similar manner (for review, see Dawson, 2004). Training was stopped after either 50,000 training iterations, or after the mean squared error on the training patterns fell below .01.
