# Supplementary material for: Artificial neural networks reveal individual differences in metacognitive monitoring of memory
Source: PLoS One. 2019 Jul 31;14(7):e0220526. doi: 10.1371/journal.pone.0220526 (PMC6668824; doi:10.1371/journal.pone.0220526)
Supplement: S3 Appendix — (DOCX) [file pone.0220526.s003.docx]

**S3. Appendix**

**Supplemental Artificial Neural Network Analysis**

The following is a supplemental artificial neural network analysis to accompany Zakrzewski, Wisniewski, Williams, and Berry (2019). In the full article, we describe how using a multilayer perceptron with 2 hidden units revealed two different clusters of older adults with different profiles for metacognitive judgments. These clusters were evident in the activities of hidden units.

It is important to confirm that the artificial neural network analyses we reported can be replicated (i.e., that conclusions do not stem from a one-off fluke in training a model), and that the results reported were not specific to the selected network architecture. To assess the former, we trained 20 additional networks in a manner identical to the network reported in the paper. Figure S1 shows hidden unit activities for young (red) and old (blue) individuals for each of these additional networks. Each trained network shows the same type of pattern as revealed in the paper where two clusters of older adults are grouped in separate areas of hidden unit space. Younger adults’ group into a single corner.


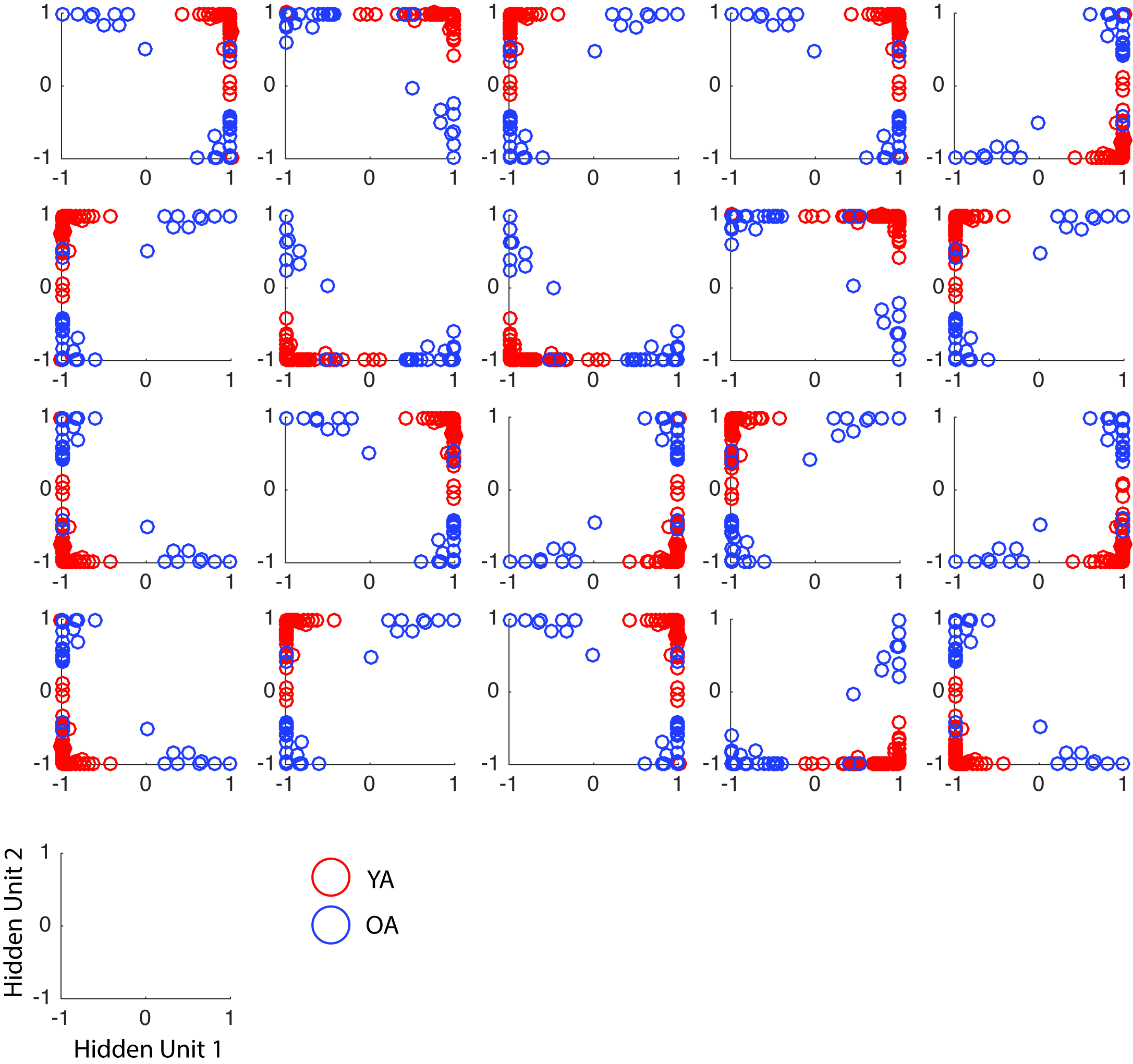


*Figure S1.* Hidden unit activities for 20 multilayer perceptrons trained to classify young adults (YAs) and older adults (OAs) in the exact manner and with the exact architecture as the artificial network reported in Zakrzewski et al. (2019). The trend for two clusters of OAs is clear for each training network.

Next, we trained a multilayer perceptron with 3 hidden units to see the if same trend could be observed when network architecture was changed. Except for the additional hidden unit, network training was identical to the network reported in the paper. Figure S2 shows activities for each individual in hidden units. Note that a similar clustering is revealed.


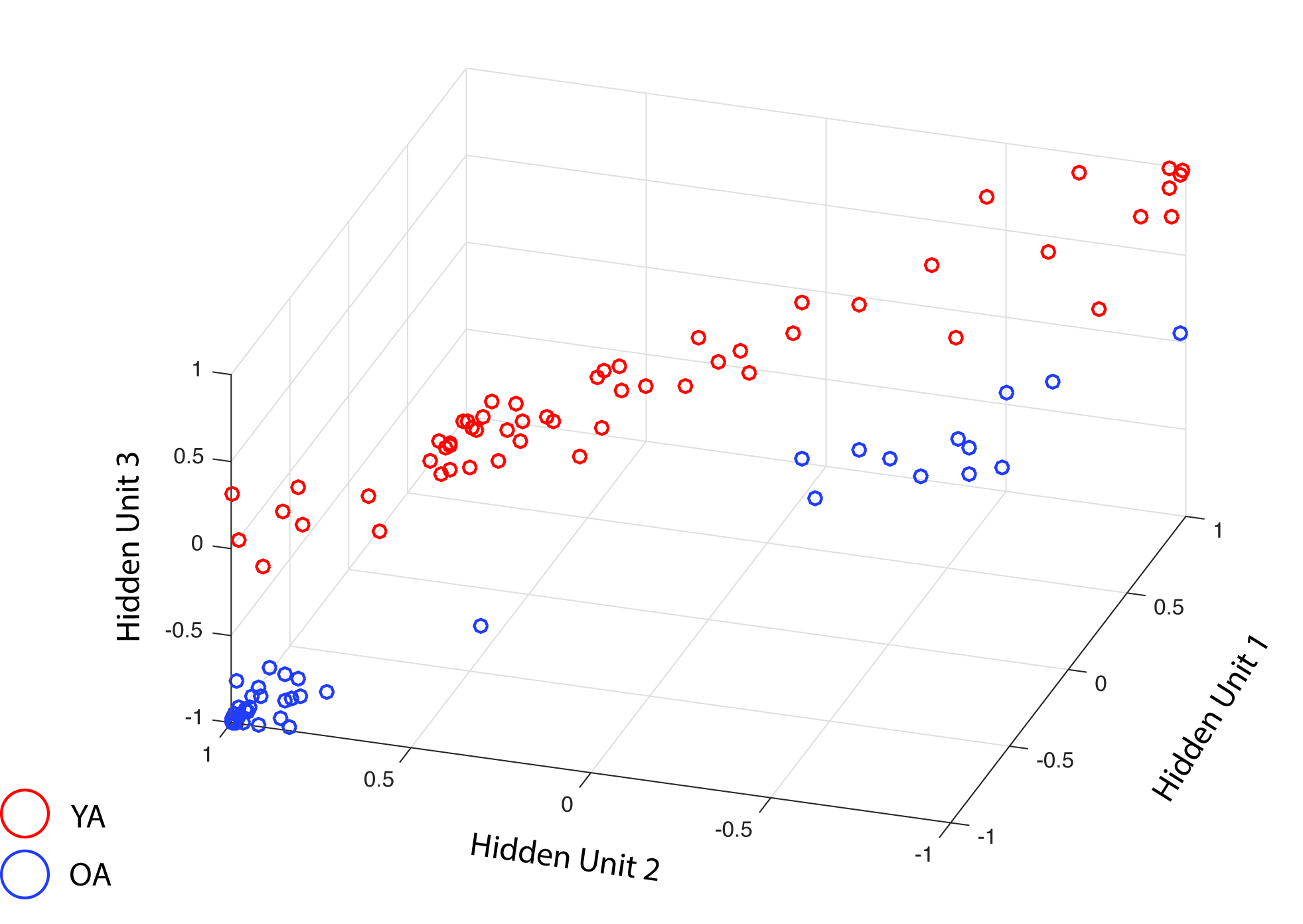


*Figure S2.* Hidden unit activities for each individual after training of a multilayer perceptron with 3 hidden units. Note the separate clusters of older adults.
