# Supplementary material for: Artificial neural networks reveal individual differences in metacognitive monitoring of memory
Source: PLoS One. 2019 Jul 31;14(7):e0220526. doi: 10.1371/journal.pone.0220526 (PMC6668824; doi:10.1371/journal.pone.0220526)
Supplement: S1 Questionnaire — Questionnaire used to collect postdictions, strategies, effort, difficulty, tiring (“fatigue”), and keeping up (“stamina”) in the “words” stimulus condition. Post-test questionnaire for names and nonwords were identical except for change in stimulus term (“word”, “name”, “nonword”). (DOCX) [file pone.0220526.s004.docx]

Post-test Questionnaire - WORDS

Instructions: To complete the scales on this questionnaire please draw a line to indicate how you rate your memory.

FOR EXAMPLE: A line in this position would indicate you thought you recognized approximately 65% of the items.

| 0% | 50% | | 100% |
| --- | --- | --- | --- |
|  | |  | |

ANOTHER EXAMPLE: A line in this position would indicate you thought you recognized approximately 40% of the items.

| 0% | 50% | | 100% |
| --- | --- | --- | --- |
|  | |  | |

1. How much did you remember in this study? Please rate your memory for each part of the experiment by drawing a line across the 0 to 100% line. 0% means ‘not remembering anything’ and 100% means ‘remembering everything’.

Individual words test:

| 0% | 50% | | 100% |
| --- | --- | --- | --- |
|  | |  | |

Word-pairs test:

| 0% | 50% | | 100% |
| --- | --- | --- | --- |
|  | |  | |

1. Did you use any tricks or strategies to help you learn the items and their pairings? If so, please describe them in the boxes:

| Individual words test |  |
| --- | --- |
| Word-pairs test |  |

1. What proportion of the time do you think your tricks or strategies were successful? Less than half? More than half? Please give a rating for each part of the experiment on a scale of 0 to 100%. 0% means ‘my strategies were not successful’ and 100% means ‘my strategies were always successful’.

Individual words test:

| 0% | 50% | | 100% |
| --- | --- | --- | --- |
|  | |  | |

Word-pairs test:

| 0% | 50% | | 100% |
| --- | --- | --- | --- |
|  | |  | |

1. How much effort did you put into the different parts of this experiment? (Please circle a number for each part of the experiment):

|  | Very little effort |  |  |  | A great deal of effort | |
| --- | --- | --- | --- | --- | --- | --- |
| Individual words test | 1 | 2 | 3 | 4 | | 5 |
| Word-pairs test | 1 | 2 | 3 | 4 | | 5 |

1. How difficult did you find this experiment? (Please circle a number for each part of the experiment):

|  | Very  easy |  |  |  | Very difficult |
| --- | --- | --- | --- | --- | --- |
| Individual words test | 1 | 2 | 3 | 4 | 5 |
| Word-pairs test | 1 | 2 | 3 | 4 | 5 |

1. How tiring did you find this experiment? (Please circle a number for each part of the experiment):

|  | Not tiring at all |  |  |  | Very  tiring |
| --- | --- | --- | --- | --- | --- |
| Individual words test | 1 | 2 | 3 | 4 | 5 |
| Word-pairs test | 1 | 2 | 3 | 4 | 5 |

7. For the YES/NO memory tests, did you have any problems keeping up with the presentation of the words and word-pairs on the screen? (e.g. you got lost and missed one/some?)

|  | I had no problems keeping up |  |  |  | I found it very difficult to keep up |
| --- | --- | --- | --- | --- | --- |
| Individual words test | 1 | 2 | 3 | 4 | 5 |
| Word-pairs test | 1 | 2 | 3 | 4 | 5 |
